# Pyrosequencing of *Haliotis diversicolor* Transcriptomes: Insights into Early Developmental Molluscan Gene Expression

**DOI:** 10.1371/journal.pone.0051279

**Published:** 2012-12-07

**Authors:** Zi-Xia Huang, Zhi-Sen Chen, Cai-Huan Ke, Jing Zhao, Wei-Wei You, Jie Zhang, Wei-Ting Dong, Jun Chen

**Affiliations:** 1 State Key Laboratory of Marine Environmental Science, Xiamen University, Xiamen, People’s Republic of China; 2 Department of Marine Biology, College of Ocean and Earth Sciences, Xiamen University, Xiamen, People’s Republic of China; Nanjing Forestry University, China

## Abstract

**Background:**

The abalone *Haliotis diversicolor* is a good model for study of the settlement and metamorphosis, which are widespread marine ecological phenomena. However, information on the global gene backgrounds and gene expression profiles for the early development of abalones is lacking.

**Methodology/Principal Findings:**

In this study, eight non-normalized and multiplex barcode-labeled transcriptomes were sequenced using a 454 GS system to cover the early developmental stages of the abalone *H. diversicolor*. The assembly generated 35,415 unigenes, of which 7,566 were assigned GO terms. A global gene expression profile containing 636 scaffolds/contigs was constructed and was proven reliable using qPCR evaluation. It indicated that there may be existing dramatic phase transitions. Bioprocesses were proposed, including the ‘lock system’ in mature eggs, the collagen shells of the trochophore larvae and the development of chambered extracellular matrix (ECM) structures within the earliest postlarvae.

**Conclusion:**

This study globally details the first 454 sequencing data for larval stages of *H. diversicolor*. A basic analysis of the larval transcriptomes and cluster of the gene expression profile indicates that each stage possesses a batch of specific genes that are indispensable during embryonic development, especially during the two-cell, trochophore and early postlarval stages. These data will provide a fundamental resource for future physiological works on abalones, revealing the mechanisms of settlement and metamorphosis at the molecular level.

## Introduction

The early development of benthic mollusks involves several fundamental developmental processes, such as spiral cleavage, body plan construction, settlement and metamorphosis, and shell formation. Studies of these processes [1]–[12] have contributed to knowledge of developmental biology, cell biology and larval ecology and have further clarified metazoan evolutionary trees. Abalones are important models for studying the early development of mollusks, specifically in the short period of time from a fertilized egg to settlement (3–5 days), and their inducibility to settlement by benthic cues [13]. Like many other spiralian lophotrochozoans, abalone embryogenesis includes spiral cleavage, mesentoblast formation and a trochophore larval stage [12]. After torsion, trochophores develop into free-swimming veligers, which are lecithotrophic and become competent after a short pelagic stage of approximately 2–4 days [14]. Competent veligers can be induced to settle and metamorphose by γ-aminobutyric acid (GABA) [15], coralline algae [11] and other environmental cues [3]. Other molecular pathways underlying the pelagic-benthic transition have also been suggested, such as adult body plan construction by *hox* genes [16]–[18], environmental morphogenetic signaling pathways [3], anticipatory pathways [2], and digestive system and shell formation [5]. Transcriptomic profiling studies on the settlement of *H. asinina* indicate that differential gene expression is widespread during the pelagic-benthic transition [19].

However, from a broader perspective, most of the molecular mechanisms underlying the early developmental processes of mollusks remain unknown except for settlement and metamorphosis. An integrated understanding of gene regulation mechanisms requires gene context and the temporal dynamics of gene expression at a global scale. Lacking global gene backgrounds has seriously retarded the further understanding of the gene regulation mechanisms of early molluscan development. Although the genomes and large-scale transcriptomes of several mollusks, such as the genome of the limpet *Lottia gigantea* (JGI) [20] and the deep-sequenced transcriptomes of the sea hare *Aplysia californica* [21], the yesso scallop *Patinopecten yessoensis* [22], the oysters *Crassostrea virginica* [23] and *C. gigas* [24], the Antarctic bivalve *Laternula elliptica* [1], the mussel *Mytilus galloprovincialis* [25] and the abalone *Haliotis midae* [26], have recently been sequenced, only a few mollusk species have had their early developmental transcriptomes sequenced or had their early developmental gene expression profiles constructed, e.g., the sea hare *A. californica* [27], [28], the snail *Ilyanassa obsolete* [29], the clam *Meretrix meretrix* [30] and the abalone *H. asinina* [19]. Thus, a substantial amount of fundamental work remains to be performed in this area.

The small abalone *H. diversicolor* is a major cultured shellfish of the south coastal areas of China. Since the late 20^th^ century, diseases, developmental dyssynchrony and a failure to settle among larvae have all frequently occurred, and the shellfish industry has been seriously impacted. To our knowledge, molecular biology and transcriptomic approaches have seldom been employed to address these problems, and almost all the publicly available ESTs for *H. diversicolor* have been generated using adults [31]. Limited developmentally related gene backgrounds have seriously inhibited both academic and industrial studies. Therefore, the deep sequencing of *H. diversicolor* larval transcriptomes will significantly enhance future studies. In the present study, seven transcriptomes from different early developmental stages of *H. diversicolor* were deep sequenced using the Roche/454 pyrosequencing platform, and 35,415 unigenes were assembled. Moreover, a reliable approach for gene expression profiling was developed, and a profile matrix was constructed and verified. An accurate gene context and global digital profile that covers early abalone stages will benefit future *H. diversicolor* larval studies.

## Results and Discussion

### 1. Experiment Design

Few *H. diversicolor* sequences were published and the next-generation sequencing platforms were at the early stage of commercialization when this study was initiated. This study would achieve two purposes: a fundamental set of unigenes and a global gene expression profile. To achieve the first purpose, 454 sequencing system was selected because it provided longer read length and even singletons could be treated as ESTs. Sequencing depth above 300,000 reads with average length of 300 bp would cover most of larva-related genes. However, to achieve the second purpose, several factors would be considered. First, sampling should cover all of the early developmental periods of the small abalone from the fertilized egg to the postlarva and developmental synchronies should be strictly controlled. Second, cDNA libraries should be separately constructed and they should be non-normalized to preserve quantitative information of gene expressions. Third, certain sequencing redundancies should be achieved to identify gene differential expressions. If sequencing redundancies were failed, additional deeper sequencing system, such as Illumina platform, would be employed. Fortunately, as described below, expression profiles of a bunch of genes were strictly constructed by statistic methods and they were confirmed to be reliable by qPCR experiments. Thus deeper sequencing was not employed in this study.

### 2. Sequencing and Assembly

**Figure 1:**
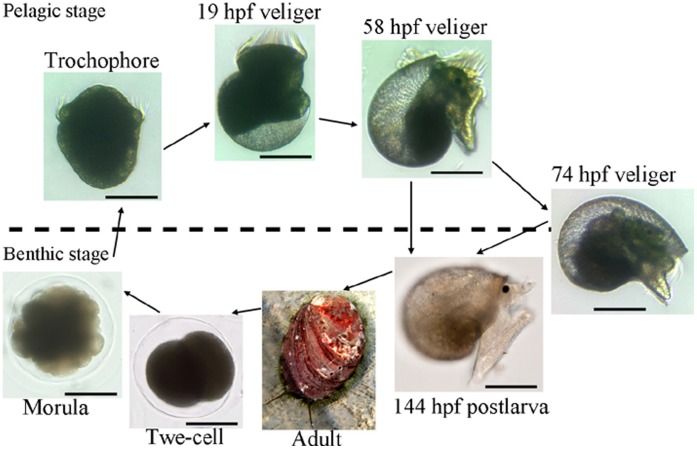
Developmental stages of the abalone *H. diversicolor* that were sampled for transcriptomic analyses. The stages include two-cell (2CELL, 0.5 hpf), morula (MORU, 3.2 hpf), trochophore (TROC, 9.5 hpf), 19 hpf veliger (19VEL), 58 hpf veliger (58VEL), late competent veliger larvae (74VEL, 74 hpf), postlarvae after 3 days settlement (144PL, 144 hpf) and adult intestinal tissue (INTE, 18 months). Scale bar = 100 µm.

**Figure 2:**
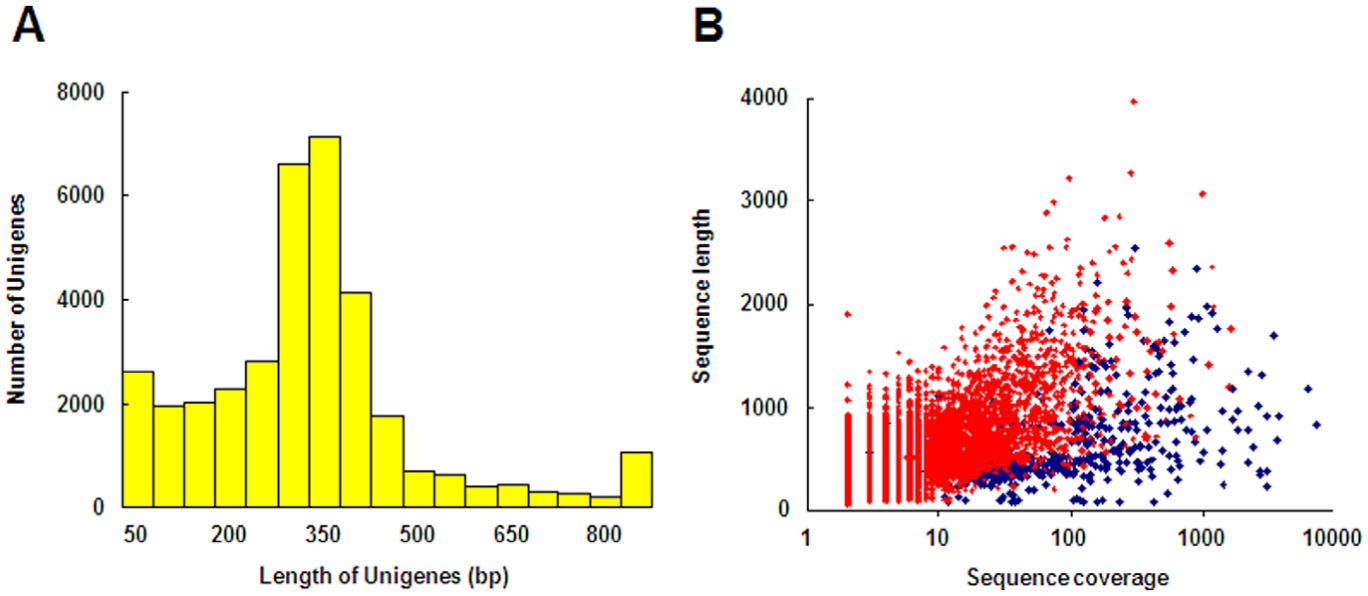
Overview of *H. diversicolor* transcriptome sequencing and assembly. (A) Size distribution of the unigenes. (B) Coverage of the assembled sequences with length distributions. The red plots represent those assembled sequences without *Bsg*I restriction sites at their 5' or 3' ends, and the blue plots represent those assembled sequences with *Bsg*I restriction sites at their 5' or 3' ends, which explains the short contigs/scaffolds with high coverages.

Non-normalized cDNA libraries were constructed from seven synchronized *H. diversicolor* embryonic/larval samples, which covered all of the early developmental periods from the fertilized egg to the postlarva. As there were few references for *H. diversicolor* transcriptomes and in order to generate scaffolds/contigs with longer length and higher quality, an intestinal sample, which was for other research purposes, also was included in sequence assembly (Figure 1). After 454 pyrosequencing, the eight transcriptomes, with 366,991 reads, were segregated from a 454 run totaling 110,136,165 bases with an average length of 300.1 bp. After trimming the adaptors and removing short reads (<50 bp), low-quality sequences and redundant reads, 307,038 high-quality reads (83.7%) were preserved for assembly. The assembly processes produced 701 scaffolds and 9,567 contigs, with 25,147 sequences remaining as singletons, resulting in a unigene collection of 35,415 sequences (Table 1). The average lengths of the scaffolds, contigs and singletons were 884 bp, 510 bp and 286 bp, respectively. The lengths of the unigenes varied from 50 to 3,966, 88.97% of which (31,508) were in the range of 100–800 bp (Figure 2A). The average contig length was rather short, which may have occurred for two reasons. First, while 48% (4,928) of the contigs/scaffolds were covered by five or more reads (Figure 2B), the sequencing depth was relatively low. The second reason may have been due to *Bsg*I restriction, which produced some short assembled sequences with high rates of coverage (Figure 2B). The quality assessment of the assembly procedure was evaluated using CD-HIT software [32], and the results demonstrated that only 83 (0.23%) sequences had significant similarities (>98% identity and >80% coverage) against the other sequences within the unigene set, indicating that the assembly was accomplished with a low redundancy. Contigs/singletons (28,044) with lengths ≥200 bp were submitted to the NCBI BioProject (ID: 86631), with nucleotide accessions JU062668 - JU071760 assigned for 9,093 contigs and JU071761 - JU090711 assigned for 18,951 singletons. The scaffolds and those contigs/singletons with lengths <200 bp are listed in Text S1.

**Table 1:** Summary of the sequencing and assembly statistics.

	2CELL	MORU	TROC	19V EL	58VEL	74VEL	144PL	INTE	Total	Average length (bp)
Raw reads	58,372	36,903	45,222	32,919	24,096	16,866	73,070	79,543	366,991	300.1
Clean reads	43,641	35,889	43,991	32,115	23,631	16,292	71,602	75,278	342,439	296.1
Non-redundant reads	38,923	33,423	41,160	30,296	22,441	14,853	67,916	58,026	307,038	300.2
Scaffolds	N/A								701	884.8
Contigs	N/A								9,567	510.3
Singletons	2,804	2,891	4,521	2,207	2,022	910	6,737	3,055	25,147	286.6
Unigenes	N/A								35,415	358.9

N/A represents that the number was not counted.

### 3. Annotatable Genes

Using the BLASTx program, sequence similarity searches of the SwissProt and NR protein databases showed that 9,513 (26.9%) unigenes had significant blast matches with E-values <1e^−5^, making them an annotatable gene set. The annotatable proportion was low; however, it was comparable to those found in other molluscan species, such as 16.8% of *H. midae* [26], 17% of *L. elliptica* [1], 24% of *R. philippinarum* [33] and 28% of *P. yessoensis* [22]. Of the scaffolds/contigs, 42.4% (4,352 sequences) had BLAST matches, whereas 20.5% (5,161 sequences) of the singletons had matches, which indicates that the abundantly expressed genes as a whole had an obvious advantage in the functional annotation. Among these annotatable sequences, 8,232 (86.5%) were >300 bp and 603 (6.3%) were >1 kb. Unique gene names for 8,331 of the sequences were found, roughly illustrating the number of genes with known functions that are expressed during the early stages and in the adult intestine of *H. diversicolor*.

**Figure 3:**
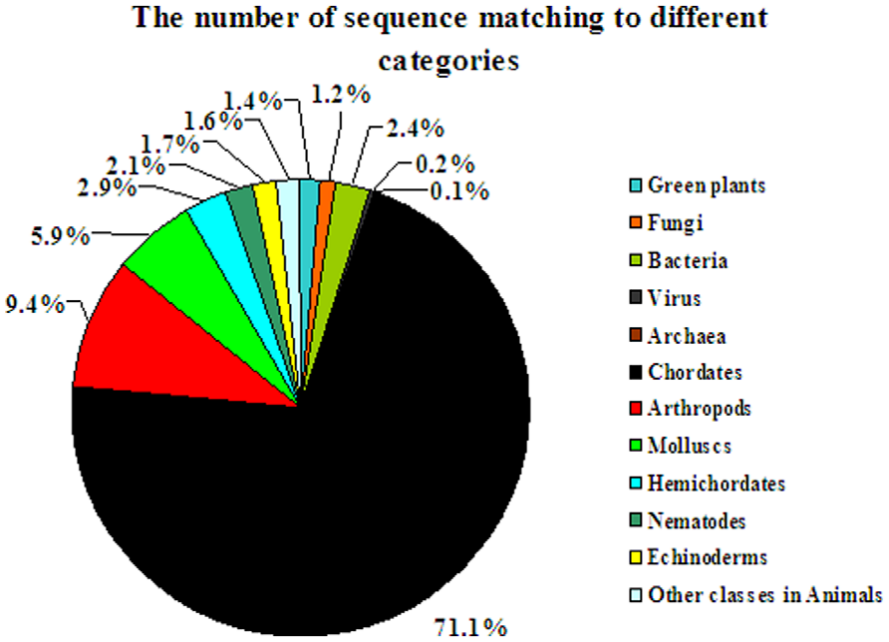
Taxonomic distribution of best match sequences with BLAST alignments to the *H. diversicolor* unigenes (E ≤1e^−5^).

Statistics on taxonomic distribution of best match species of annotatable unigenes was summarized in Figure 3. As shown in this figure, 7,915 sequences (96.6%) were matched to animals and the other 454 sequences (5.4%) originated from plants, fungi, bacteria, archaea and viruses, clearly indicating that contamination from environmental organisms during sample preparation was very low. Apparently, a majority of the annotated sequences were matched to the Chordata, which was consistent with *A. californica* [27], *H. midae* [26] and *M. meretrix* [30]. It could be reasoned that functional studies have been much more intensely performed and thus have provided significantly more functional data on the Chordata category. However, only 5.9% of the sequences best matched to molluscan species, indicating that functional annotation efforts with a high efficiency are necessary for molluscan species.

### 4. Gene Ontology Statistics

**Figure 4:**
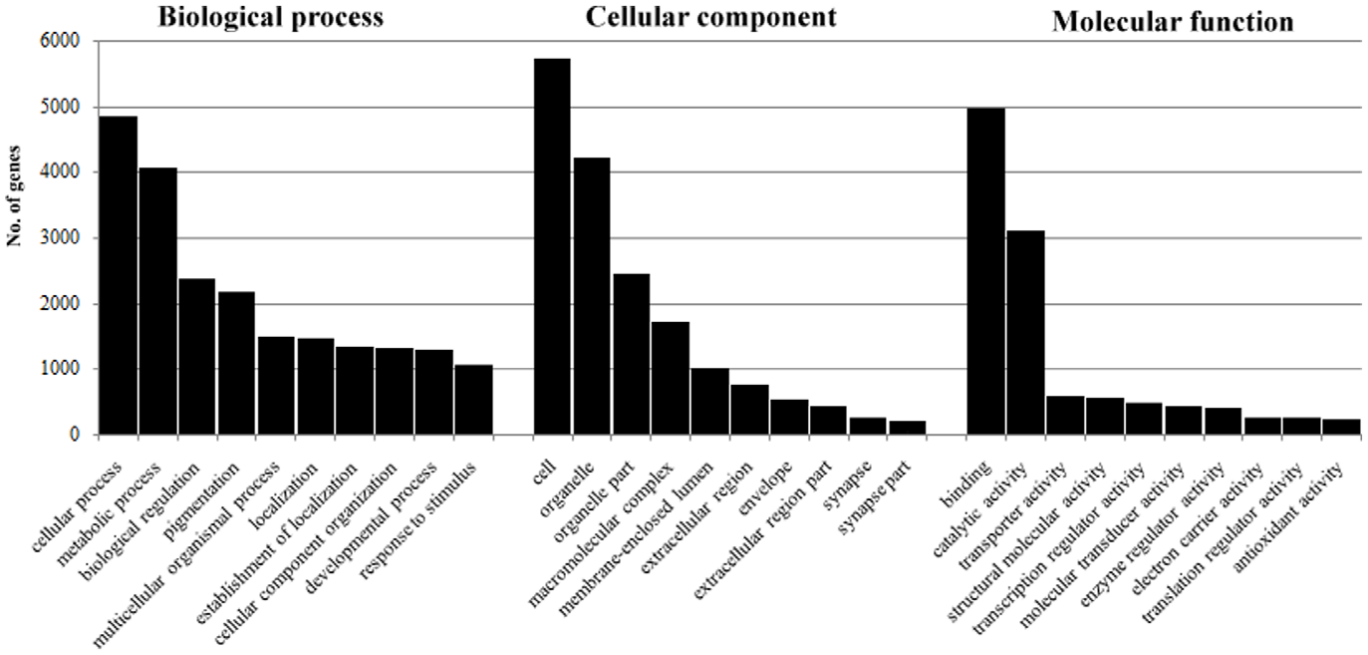
Gene ontology (GO) annotation of the *H. diversicolor* larval transcriptome. The top 10 gene ontology (GO) categories under Biological process, Cellular component and Molecular function for the early developmental transcriptomes of *H. diversicolor.*

Among the 7,566 unigenes assigned GO terms (Table S1), 6,745 contained embryonic/larval reads and thus were purified from an adult context. Of the purified embryonic/larval unigenes, 6,370, 6,410 and 6,273 sequences were successfully assigned to the Biological process, Cellular component and Molecular function categories, respectively. Within each of these three main categories, genes annotated with cellular process, cell and binding were the most abundant, respectively. The top 10 second-level terms for these three main categories are displayed in Figure 4. Important cell procedures related to early development were roughly indicated; for example, 159 (2.1%) genes are involved in cell motion, 236 (3.1%) genes are involved in cell proliferation and 157 (2.1%) genes are involved in locomotion.

### 5. The most Abundantly Expressed Sequences

An outline of the most active biologic processes in the embryo/larva was roughly sketched using the 20 most highly expressed genes (Table 2). These genes are closely related to energy metabolism, the cellular skeleton, protein metabolism and other basic processes that the organism requires. In Table 2, several genes that may be involved in cellular energy metabolism, such as cytochrome c oxidase subunits (1, 2 and 3), ATP synthase subunit a, NADH-ubiquinone oxidoreductase subunits (1, 4, 5 and 6) and cytochrome b, are indicated. High expression levels of these genes ensure energy transmission and transformation during *H. diversicolor* embryogenesis. Interestingly, we also discovered that four genes might function in the process of actin cell skeleton metabolism, including thymosin-beta, profilin, actin and actin-2. The active turnover and reconstruction of the actin skeleton indicate that cellular locomotion and cell shape changes are very dramatic in the abalone larva. In addition, these phenomena are highly consistent with those of *A. californica* larva, in which energy metabolism and cell-skeleton-related genes also occupy a high proportion (11/30) of the most abundantly expressed genes [27]. Further, there are seven genes that overlap between the two groups of highly expressed genes, including ATP synthase subunit a, cytochrome b, NADH-ubiquinone oxidoreductase chain 1, cytochrome c oxidase subunits (1, 2 and 3) and actin, likely indicating that Gastropoda species share some basic bioprocesses during early development.

**Table 2:** The top 20 highly expressed sequences with their associated BLAST matches (E-value<1e−5).

Seq ID	Best Match	Length (bp)	E-value	*Expression in larvae	Description	Species
JU063545	O78682	848	1E-17	29,084	Cytochrome c oxidase subunit 2	*Carassius auratus*
JU064608	Q34941	326	4E-37	24,371	Cytochrome c oxidase subunit 1	*Lumbricus terrestris*
JU064611	Q34943	929	9E-19	14,044	Cytochrome c oxidase subunit 3	*Lumbricus terrestris*
JU071714		1,696		13,940		
JU071754	P42678	919	5E-12	12,434	Protein translation factor SUI1 homolog	*Anopheles gambiae*
JU063890	P33248	382	1E-7	11,305	Thymosin beta-12	*Lateolabrax japonicus*
JU063381	ABK21482	1,320	2E-17	11,177	Unknown	*Picea sitchensis*
JU063200	AAX11341	346	7E-9	11,000	developmentally regulated vdg3	*Haliotis asinina*
JU063900	P34875	1,014	7E-33	10,402	Cytochrome b	*Sphyrna tiburo vespertina*
JU071628	Q34946	785	1E-29	8,807	ATP synthase subunit a	*Lumbricus terrestris*
JU071629	Q34947	1,349	4E-114	8,751	NADH-ubiquinone oxidoreductase chain 5	*Lumbricus terrestris*
JU071577	O47478	451	6E-86	8,675	NADH-ubiquinone oxidoreductase chain 6	*Loligo bleekeri*
JU064489	P53486	789	1E-6	7,482	Actin, cytoplasmic 3	*Takifugu rubripes*
JU062677	ABY87349	1,763	2E-87	6,594	Profilin	*Haliotis diversicolor*
JU064614	Q37546	1,202	5E-26	6,104	NADH-ubiquinone oxidoreductase chain 1	*Lumbricus terrestris*
JU071627	Q34048	1,757	2E-138	4,896	NADH-ubiquinone oxidoreductase chain 4	*Ceratitis capitata*
JU070508		1,973		4,787		
JU062954	Q9U639	2,357	1E-7	4,606	Heat shock 70 kDa protein cognate 4	*Manduca sexta*
JU071733	C7G0B5	1,979	8E-27	4,229	Aragonite-binding protein Pif	*Pinctada fucata*
JU063675	P10984	1,411	7E-23	4,072	Actin-2	*Caenorhabditis elegans*

The asterisk represents the read number of each gene per 1,000,000 reads of each catalog.

In addition to these well-known genes, two Mollusca-specific genes, developmentally regulated *vdg3* and the aragonite-binding protein Pif, were also highly detected. Developmentally regulated *vdg3* was first identified as a highly expressed gene in the digestive glands of *H. asinina* veligers and has been speculated to regulate the formation of the juvenile digestive system [4]. The aragonite-binding protein Pif plays a key role in the formation of the nacre of the molluscan shell [34]. Thus, our gene data will significantly improve studies related to mollusca-specific bioprocesses.

We also noticed that three of the twenty genes have unknown functions and that 2 of these genes (JU071714, JU070508) have no blast matches against the SwissProt, NR or TrEMBL protein databases, demonstrating that some fundamental developmental processes of molluscan species still remain unknown.

### 6. Differential Gene Expression Profiles

In addition to obtaining the context of embryonic/larval genes, another major aim of this transcriptomic study was to construct a global gene expression profile and thus be able to globally identify genes participating in important early developmental bioprocesses, especially settlement and metamorphosis. After counting and normalizing the non-redundant reads that matched each scaffold/contig in each sample, we constructed a table containing the relative expression levels of all the scaffolds/contigs from all the samples. Genes that were highly identified by 454 sequencing were considered reliable. Among these genes, those that matched a total of 16 physical non-redundant reads were included in Table S2 (1491 genes). Some of the important information from the gene expression profile is summarized below.

#### Verification

**Figure 5:**
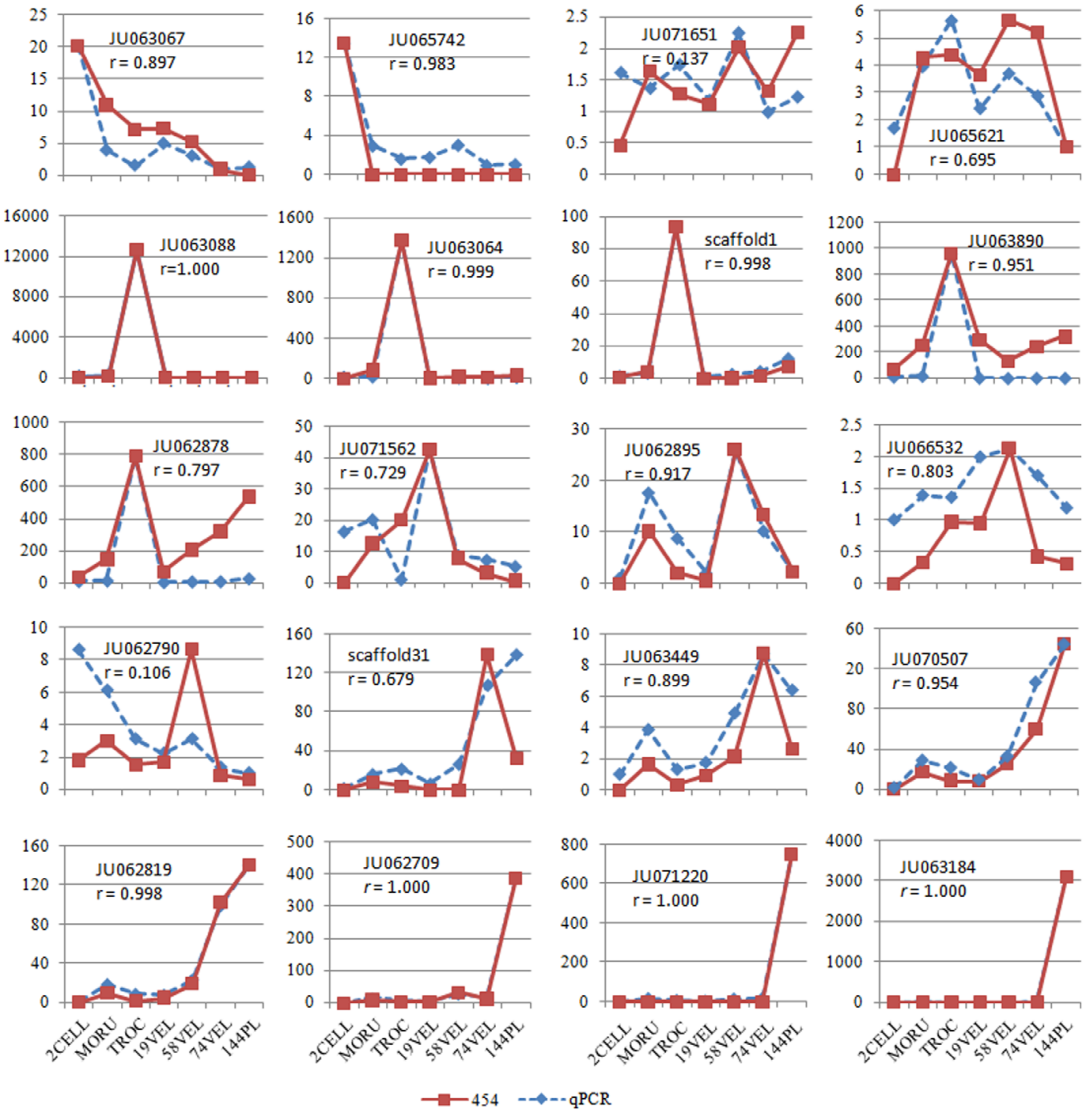
Validation of 20 temporal dynamics by qPCR. For the qPCR data, the lowest expression level of each gene was set as 1, and the other expression levels are indicated as the fold-change relative to it. The 454 expression levels of each gene were normalized to the 454 data for *OAZ1*/*YB1* and rescaled to the qPCR scales. Abbreviation: *r*, Pearson’s correlation coefficient.

The temporal dynamics of 29 scaffolds/contigs were examined using real-time PCR (qPCR). The first part of the verification was involved evaluating the stably expressed genes during the early developmental stages by using 9 genes. Y-box protein 1 (*YB1*), ornithine decarboxylase antizyme 1 (*OAZ1*) and eukaryotic translation initiation factor 5A (*EIF5A*) qualified as internal control genes (ICG) after such an evaluation [35]. The second part of the verification was to calibrate the qPCR relative expression levels of another 20 scaffolds/contigs by applying the reliable ICGs and to then evaluate the correlations between the two types of temporal dynamics using Pearson’s correlation test (Figure 5). This test was used to indicate whether the correlations were relatively high by setting *r* >0.75 as a strong correlation. Then, 75% (15/20) of the temporal dynamics of the 454 profile matrices were deemed reliable. When the threshold was lowered to *r* >0.6, 90% (18/20) of the temporal dynamics of the matrices were deemed reliable. JU071651 and JU062790 had far larger deviations, which might have been due to systemic or experimental errors of the qPCR or 454 sequencing method or due to physiological differences between the two sampling batches. Interestingly, the two genes had relatively low expression fluctuations; thus, we speculated that the expression profiles with notable fluctuations were more reliable. Compared to microarray data [19], our profiles demonstrated higher correlations to the qPCR. We reasoned that two elements impacted these results. First, our investigated scope was much wider than that of Williams *et al.,* and thus, our gene expression fluctuations were more dramatic and more reliably detected. Second, compared to a microarray method, a sequencing method is easier to quantify after applying a carefully designed transcriptomic sequencing strategy and a strict bioinformatics pipeline.

#### Cluster analysis of gene expression

**Figure 6:**
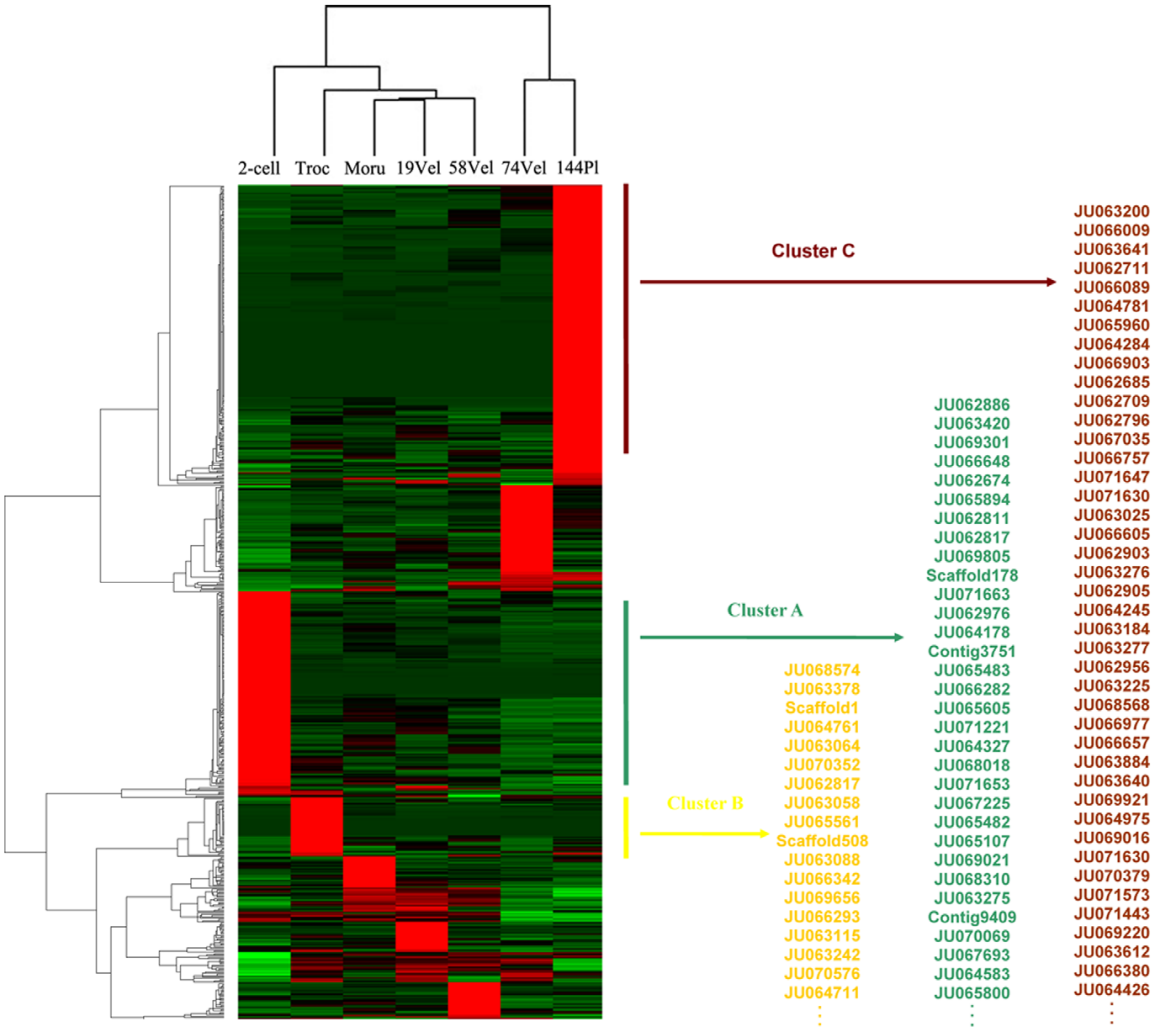
Clusters of the expression levels of the candidate genes. Dendrograms of the gene expression patterns for the 636 genes assigned 2 or more stars are shown. The clustering indicates similar expression patterns among the developmental stages (x-axis) and among the genes (y-axis). The bar color reflects the gene expression level from green (low) to black (medium) to red (high). The partial gene names used for the analysis, whose expression levels were extremely high during the two-cell, trochophore and 144 postlarval stages, are displayed in the chart. The gene IDs in these three clusters are listed in Table S3.

As described in the Methods section, the expression profiles of 636 genes were deemed reliable and hierarchically clustered, and these are displayed in Figure 6. When viewed from the x-axis of the dendrogram, the developmental stages form two distinct clusters. The two-cell, morula, trochophore, 19 hpf veliger and 59 hpf veliger stages were aggregated into one cluster, and the late competent veliger larvae (74VEL) and postlarvae (144PL) stages were aggregated into the other cluster. This type of clustering pattern is consistent with that of *H. asinina* [19] and also indicates the validity of anticipatory development, which refers to the phenomenon that some juvenile structures are formed before the larvae actually undergo the pelagic-benthic transition [2] and also indicates that the later competent larvae accumulate more of the transcripts required for metamorphosis than the younger larvae. The former dendrogram cluster indicates that the cluster order is not consistent with the developmental stage and that the trochophore is distinct from the earlier and later stages (Figure 6). These results imply that gene expression is specific in the trochophore, the stage when larvae are hatching from their egg envelopes and beginning to swim freely.

When viewed from the y-axis of the dendrogram, gene clustering indicates that each stage possesses distinct genes that are highly expressed. These highly expressed genes predominantly appear during the two-cell, trochophore, 74 hpf veliger and 144 hpf postlarval stages. Deeply investigating concrete gene expressions patterns will facilitate the discovery of key genes regulating the early developmental processes of abalones. Among the seven developmental stages of *H. diversicolor*, we have a profound interest in understanding the gene transition process from the two-cell to trochophore to postlarval stages. The two-cell stage is the beginning of the embryonic development of abalones, and the trochophore stage is related to hatching and the transition to the free-swimming phase. Finally, the postlarval stage is the end of the free-swimming phase and the beginning of benthic life. Correspondingly, three clusters (Figure 6, Table S3) were extracted from the matrix. Most of these genes have unknown functions, and those genes with important known functions are summarized below.

*Cluster A.* The transcript abundances of the genes in this cluster were highest during the two-cell stage and quickly dropped during the following stages. These transcripts are likely of maternal origin because it is biologically inefficient to rapidly express a transcript and then rapidly deplete it within a short period of time such as three hours. The depletion rates of these transcripts are very high. For example, the abundance levels of JU062817 and JU065482 mRNA dropped 78-fold and 57-fold, respectively, during the period of time from the two-cell stage to the morula stage. The rapid depletion of several other mRNAs may also be important for the initiation of embryogenesis, including JU062817, which is highly similar to DnaJ, a protein that functions as a repressor of cell division and cell differentiation during embryogenesis [36]–[38], and JU065482, which is similar to the *Drosophila* gene *brat*, a translational repressor through the recruitment of protein–protein interactions with the Nanos/Pumilio/RNA complex [39], [40]. We speculate that the main function of these genes is to preserve the egg cell from dividing and differentiating and that this lock system must be rapidly removed during subsequent embryogenesis processes.

*Cluster B.* In this cluster, genes were highly expressed during the trochophore stage. Compared to other developmental periods, JU063088 expression was increased over 100-fold during this stage. JU063088 is highly similar to collagen alpha-4 (VI) chains (COL6A4), which co-assemble with other endogenous collagen chains to form trimeric collagen VI molecules and secrete from cells to form abundant and structurally unique ECM components [41]. Some cargo-transport-related genes, such as VAP-33 (JU064429) and β-thymosin (JU063890), also exhibited their highest expression levels during this stage. Thus, we propose that the highly expressed COL6A4 protein and other structural components are cargo transported outside of cells and then form a collagen shell to protect the larval body after hatching. Scaffold508 displayed an abundance of at least 30-fold greater than other stages during this period. This protein is highly similar to members of the low-density lipoprotein receptor-related protein family (LRP), which are large multifunctional clearance receptors that mediate diverse cellular processes ranging from cargo transport to signaling [42]–[44]. LRP also plays important roles during development [45], [46]. However, there are currently no reports available on its function during molluscan embryogenesis. Thus, this gene expression profile provides valuable information for further studies.

*Cluster C.* This cluster contains genes that are highly expressed during the postlarval stage. Some of these genes are related to signaling, such as fibropellin-1 (JU062709), EFCBP (JU071630) and SARP-19 (JU063184). Fibropellin-1 is also known as epidermal growth factor-related protein 1. This protein was first discovered in sea urchin embryos and has been demonstrated to function during developmental growth. Because of its role in specific signaling transduction pathways, fibropellin-1 loss leads to development defects [47], [48]. The SARP-19 of the marine snail *Littorina littorea* contains two putative EF-hand domains and is abundantly expressed under anoxic exposure [49]. The increased expression of signaling genes indicates that some gene regulation networks are initiated. Consistently, genes related to histogenesis programs, such as the digestive system and immune system, were also identified. As one of the 20 most abundant transcripts and the most abundant transcript in postlarva stage (Table S2), developmentally related *vdg3* (JU063200) was expressed at least 29-fold higher during this stage than in any earlier stage. Its expression pattern is consistent with its *H. asinina* counterpart, which is definitively expressed in the digestive gland of early postlarvae [4], [19], indicating the gene relates to either gut morphogenesis or digestion. Based on its sequence, JU064245 is likely an enteropeptidase, which is a class of enzymes that catalyze the conversion of trypsinogen to trypsin and, in turn, activate other proenzymes (trypsin, chymotrypsin and carboxypeptidase A) [50]. A high expression level of this enzyme indicates that the digestive system is active. Immune-related genes, such as defensin (JU063277) and GM2-AP (JU062796), were also identified during this stage. Defensins are small, arginine-rich cationic proteins that are active against microbes by binding to and embedding in the cell membrane, which forms pore-like membrane defects that allow for the efflux of essential ions and nutrients. The expression pattern of these genes in *H. diversicolor* was similar to that of *M. meretrix*, in which defensin mRNA begins to appear when the larvae begin to feed [30]. We speculate that larvae are more exposed to pathogens in their environment when acquiring nutrients, and thus, a higher immune response is stimulated. Of interest, several genes involved in cell adhesion or ECM, such as MERP1 (JU066089), ADAM-TS16 (JU071647), DZ-HRGP (JU063025) and mucin (JU066605), were in this cluster. MERP family members are involved in cell adhesion [51], and ADAM family members are membrane-bound cell-surface glycoproteins with numerous functions in cell physiology [52]. The increased expression of ECM-related genes implies that the differential gene expression levels of these tissues are dramatic and that the ECMs function as chambered structures.

### Conclusion

By jointly applying a carefully designed transcriptomic sequencing strategy, a strict bioinformatics pipeline and rigorous quality controls, we obtained both a global gene context and global gene expression profile of the early development of *H. diversicolor* with a high degree of quality. Similar to other molluscan transcriptomes, over 70% of the genes of the *H. diversicolor* transcriptomes were of an unknown function. The annotation of these genes will require highly efficient functional studies. Our global gene expression profiles contain the precise temporal dynamics of more than six hundred genes. This is the first digital profile that covers the entire early developmental stages of a mollusk. Using cluster analyses of gene expression, we have listed and analyzed some of the specific genes that are highly expressed during the two-cell, trochophore and postlarval stages. Therefore, we propose some bioprocesses that have not yet been verified, such as a lock system in mature eggs, a collagen shell of trochophore larvae and chambered ECM structures in early postlarvae. In terms of the global gene expression background of the entire early developmental process, key genes related to important early developmental processes can be identified with a higher degree of accuracy. Thus, our data provide valuable clues for further functional studies.

## Materials and Methods

### 1. Sample Collection

*H. diversicolor* embryos/larvae were from the Hongyun abalone farm (Zhangzhou, Fujian). Following the procedure of You *et al*. [53], adult abalones, 20 females and 5 males, were induced to spawn for artificial fertilizations. The culture temperature was 23–25°C. Seven embryonic/larval samples were collected: two-cell (2CELL, 0.5 hpf), morula (MORU, 3.2 hpf), trochophore (TROC, 9.5 hpf), 19 hpf veliger (19VEL), 58 hpf veliger (58VEL), late competent veliger larva (74VEL, 74 hpf), and postlarvae after 3 days settlement (144PL, 144 hpf). In this sampling, the hatching of trochophores occurred at approximately 9.5 hpf, and settlement began at 58 hpf. Except for 144PL, the developmental synchronies were monitored using a microscope to ensure that over 80% of the larvae in each sample were at the same developmental stage. 144PL contained postlarvae that settled at 58 hpf ∼ 82 hpf. Each collected sample was immediately washed with ddH_2_O and preserved in TRIzol (Invitrogen, USA). To enlarge the reference gene library and for future data comparisons, intestinal tissue was also sampled from an 18-month-old adult. Prior to RNA isolation, the samples were stored at −80°C.

### 2. RNA Extraction and the cDNA Procedure

Each sample (140∼260 mg) was used for RNA extraction. Total RNA was extracted using a TRIzol kit (Invitrogen, USA), and the integrates of the total RNA were evaluated by running 1.2% agarose gels. The mRNA was further purified using a MicroPoly(A) Purist kit (Ambion, USA). Non-normalized cDNA libraries were prepared as previously described [54] with some modifications. First and second cDNA strands were synthesized from 50–200 ng of mRNA using a SMART™ PCR cDNA Synthesis kit (Clontech, USA). However, the 3′ SMART™ CDS Primer II A primer was replaced with a CDS/*Bsg*I primer (5′- ATTCTAGAGGCCGAGGCGGC*GTGCAG*TTTTTTTTTTTTTTTTTTTVN -3′), which had a *Bsg*I endonuclease recognition site for future polyA-tail removing. Double-stranded cDNA (5 µg) in 300 µL was sonicated for 60 seconds (with cycles of 3 seconds on and 3 seconds off) on ice using a Sonifier S450-D sonicator (Branson, USA) at 10% power. After *Bsg*I digestion and end-polishing, the cDNA fragments were ligated to MID-modified Titanium adaptors (454 Life Sciences) and amplified using Titanium A/B primers (454 Life Sciences) with 12–15 cycles, followed by size fractionation by 1.2% agarose electrophoresis. The cDNA fragments within the size range of 300–600 bp were excised from the gel and purified using a Qiagen Gel Extraction kit. After the procedure, each cDNA library was barcoded using one specific MID. The libraries were quantified and pooled, and emulsion PCR was performed. The libraries were then sequenced using a 454 GS FLX Titanium system at Majorbio Biotech (Shanghai, China). A Perl script was written to identify the MIDs and to segregate the libraries. This script is freely available by contacting the authors.

### 3. 454 Sequence Assembly and Annotation

The segregated reads from the different libraries had their adaptors trimmed, short reads removed (<50 bp) and low-quality areas filtered, as described by Meyer *et al*. [54]. The probability that sonication would result in the same fragments was very low; thus, most of redundant fragments must have resulted from non-balanced amplification during the library construction and emulsion PCR steps. Therefore, redundant reads containing the same sequences and with the same lengths in each library were removed using CD-HIT [32]. After this procedure, the resultant non-redundant clear reads were combined to assemble a reference library. The assembly was performed in four steps. Step 1: The reads were assembled by using Newbler v2.3 (Roche) for the cDNA project and without the –rip option to preserve most of the branch structures. Each isogroup, which contained putative alternative transcript isoforms (isotigs) from one gene, was manually checked to remove *Bsg*I artifacts. Multiple isotigs in each isogroup were manually condensed into one sequence with “n” to mark the putative alternative splicing sites. This condensing was used to facilitate the further construction of the gene expression profile. The original isotigs were preserved for functional annotation. Step 2: DNA dragon assembly software was used to recover and to assemble reads that were not previously assembled into isotigs by Newbler. Step 3: There was a probability that two or more reads came from the same transcript but contained no detectable overlaps. To connect these reads, scaffolds were further joined according to a shared similarity with known proteins [54]. Step 4: Sequence redundancy was further assessed and reduced by CD-HIT, which removed sequences that had 98% or higher identity to the longest sequence in that contig. It should be noted that Newbler has specific terms to describe assembly outputs. However, the outputs were further assembled by later procedures, and the Newbler attributes of the sequences were difficult to trace. To make the procedure more straightforward, we defined a singleton as a non-redundant clear read that has no overlap or connection with any other sequence, a contig as a consensus sequence of a set of overlapping reads, and a scaffold as a joined sequence of two or more assembled sequences that has no overlap while sharing similarity with a known protein. These three categories composed the final assembled unigene set.

The determined unigenes were annotated based on sequence similarity against the SwissProt and GenBank non-redundant (NR) protein databases using the BLASTx program. The Gene Ontology (GO) terms were assigned via Blast2GO software [55] using BLAST matches to the SwissProt and UniProt-TrEMBL protein databases with an *E*-value threshold of 10^−5^.

### 4. qPCR Experiments

Template cDNA preparation and primer quality controls were performed as internal control gene (ICG) evaluations [35]. In brief, another batch of embryonic/larval samples was prepared and collected from the same abalone farm. The water temperature was strictly controlled, and the overall developmental processes for the larvae were identical to the former sampling, except that the hatching peak for the trochophores occurred approximately 20 minutes earlier. Full-length cDNA libraries were constructed using the same kits and conditions described for the transcriptome sequencing, except that the CDS/*Bsg*I primer was replaced with the 3′ SMART™ CDS Primer II A primer for the synthesis of the first- and second-strand cDNA. The accuracies and specificities of the primer pairs for the qPCR were evaluated by PCR using a cDNA mixture as the template. Those primer pairs produced a single band, and the correct amplicon lengths were further used for the qPCR. The seven early developmental cDNAs were diluted to 0.1 ng µL^−1^ and used as templates. The qPCR experiments were performed using a Rotor-Gene 3000 Real-time PCR instrument (Corbett). Each reaction consisted of 1 µL of 5 µM forward primer, 1 µL of 5 µM reverse primer, 1 µL of template, and 12.5 µL of 2×*TransStart Green qPCR SuperMix* (Transgene Biotech, Beijing, China) added to ddH_2_O (final volume 25 µL), and was repeated three times. The PCR conditions were as follows: 95°C for 3 minutes, (95°C for 10 seconds, 55°C for 15 seconds, 72°C for 15 seconds) × 45 cycles. The relative expression levels of each gene for the different developmental stages were normalized to the Y-box protein 1 gene (*YB1*) and ornithine decarboxylase antizyme 1 gene (*OAZ1*), which have been verified as reliable ICGs in previous studies [35]. To evaluate whether the relative expression levels of the qPCR and 454 corresponded, the 454 data was also normalized using the 454 expression data for *YB1*/*OAZ1* and then compared with the qPCR data using Pearson’s correlation tests in Microsoft Excel.

### 5. Construction of the Gene Expression Profile

For each library, the non-redundant reads were matched to the contigs/scaffolds using BLASTn with an E-value <1e^−30^. If a read had multiple hits, only the best-hit contig/scaffold was recorded. The number of reads that registered as hits with each contig/scaffold for each library was counted. The relative expression level of a contig/scaffold in a sample was then normalized as (the read count of a scaffold/contig)/(the total non-redundant read number for the library)×one million. The relative expression levels were then organized into an Excel table with the gene order indicated vertically and the stage order indicated horizontally. The ENCODE Consortium recently released strict criteria to aid in the construction of transcriptional profiles obtained using RNA-Seq technologies [56]. However, our sequencing depths were obviously below the criterion of 30 M reads for each library. In an attempt to balance our ambitions to make the best use of the data and the basic requirements for statistical confidence, some mathematical statistics rules were applied to filter or mark the data. First, the counts for each gene during the seven early stages were summed, and low-count genes were filtered using the σ rules of mathematical statistics; it is commonly accepted that high abundant genes will be less affected by the sequencing depth than the low-abundance genes [57]. The underlying theory is that, in the Poisson statistics of random events, observed events with higher counts will have smaller measurement errors in setting the confidence interval, which is described by the mathematical formula below.



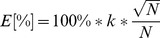
(1)
[58]

where E [%] is a relative statistical error, N is an observed count number, and k sets the confidence interval, e.g.,k = 1, 1σ → 68.3%, where σ is the standard deviation;k = 2, 2σ → 95.5%;k = 3, 3σ → 99.7%.

When setting the confidence interval as 1σ and E [%] as 25%, the formula (1) derives the cut-off count (N) as equal to 16. The physical meaning is that if an observed count was larger than 16, then the true count has a 68.3% probability of being located in an interval of (1±25%)*N at its widest. Such loose constraints preserved most of the informative data.

Second, confidence levels were marked for those genes remaining in the table. The temporal dynamics of each gene are composed of seven expression data points. If each of the data points was derived from high counts, then the temporal dynamics should have a high confidence level. The formula below was deduced to mark such a confidence level.
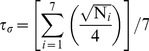
(2)


The physical meaning of the operator is that if each of the seven counts (N_1_∼N_7_) of a gene was 16, then the confidence τ_σ_ of the temporal dynamics is 1-star.

However, gene profiles with dramatic expression level changes were more reliable. To find these genes, Q-tests [59] for outlier detection were applied.
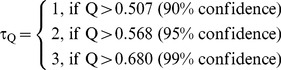
(3)


Here, 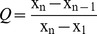


The physical meaning of this evaluation is that if one expression data point appears to deviate from the other 6 data points for the same gene with 90%, 95% or 99% confidence, then the gene would be valued as 1-, 2- or 3- star, respectively.

After applying the evaluation tests from above, the selection of the candidate genes used for the analysis of gene expression was based on the star values of the genes.

### 6. Cluster Method of Gene Expression

Cluster analyses of gene expression were performed on the normalized, filtered sequences to identify genes whose expression levels varied significantly among the different developmental stages. To make our result more reliable, we selected those genes valued with more than 2 stars in total as candidates (636 genes) for the analyses using Cluster 3.0 software. In our expression data, some of the gene expression values were zero for certain stages. However, this result did not indicate that those genes were not expressed during those stages but that their expression levels were too low to be detected. Therefore, we exchanged this value with the fifth of the smallest values in the expression data (0.0002). Before clustering, the raw expression data were centered and normalized to prevent large variations for each value. The expression data were centered three times so that the median of the values in each raw dataset was zero, and the data were also normalized five times so that the sum of the squares of the values in each row was 1.0. The hierarchical clustering algorithm used was based on the centroid linkage, and a heat map dendrogram was generated for significant genes through the combination of a gene tree and a developmental stage tree to visualize the relationships among the different developmental stages with regard to gene expression. In addition, TreeView software was used to view the dendrogram.

## Supporting Information

Table S1 Information for the GO annotations of the unigenes.
(XLS)

Table S2 Expression data of the 1491 genes with total physical counts larger than 16.
(XLS)

Table S3 Gene IDs in clusters A, B and C.
(XLS)

Text S1 Scaffolds and contigs/singletons with lengths shorter than 200 bp.
(TXT)
